# Regulatory complexity and therapeutic targeting of the necroptosis network

**DOI:** 10.3389/fimmu.2026.1824460

**Published:** 2026-04-30

**Authors:** Lipan Niu, Fengxia Liu, Yuxin Zhao, Jiangtao Li, Hui Wang

**Affiliations:** 1 School of Basic Medical Sciences, Xinjiang Medical University, Urumqi, Xinjiang, China; 2 Xinjiang Key Laboratory of Molecular Biology of Endemic Diseases, Urumqi, Xinjiang, China; 3 Henan Key Laboratory of Immunology and Targeted Drugs, Henan Medical University, Xinxiang, Henan, China; 4 Henan Collaborative Innovation Center of Molecular Diagnosis and Laboratory Medicine, School of Medical Technology, Henan Medical University, Xinxiang, Henan, China

**Keywords:** inflammatory, necroptosis, RIPK1/RIPK3/MLKL, TRIF/RIPK3/MLKL, ZBP1/RIPK3/MLKL

## Abstract

Necroptosis, a regulated form of necrotic cell death governed by the RIPK1-RIPK3-MLKL axis, is critically involved in host defense, inflammatory responses, and the pathogenesis of diverse diseases. Given the expanding complexity of its signaling networks and their context-dependent outcomes, a synthesized overview is essential. This review aims to: (1) delineate both canonical and non-canonical pathways of necroptosis induction; (2) elucidate the multifaceted regulation of its core executors (RIPK1, RIPK3, MLKL) by post-translational modifications and epigenetic mechanisms; and (3) analyze the intricate crosstalk between necroptosis and other cellular processes, including apoptosis, autophagy, and metabolic pathways. The subsequent analysis will evaluate how this sophisticated regulatory architecture poses challenges while unveiling novel therapeutic vulnerabilities. Finally, emerging translational strategies that target necroptosis in inflammatory, neurodegenerative, and ischemic conditions are discussed, and propose future directions to bridge mechanistic discoveries to clinical applications. Notably, beyond its pathogenic roles, necroptosis also functions as an essential host defense mechanism against viral infection and represents a promising therapeutic strategy for eliminating apoptosis-resistant cancer cells, highlighting its context-dependent dual nature.

## 1 Introduction

Necroptosis is a regulated form of programmed lytic cell death that is typically activated upon inhibition of apoptotic signaling (1–3). Initially recognized as a host defense mechanism against viral infection, it has since emerged as a crucial pathogenic driver across a wide range of disorders, including inflammatory, cardiovascular, neurodegenerative, and malignant diseases (4–6). Paradoxically, necroptosis can also exert protective roles in certain contexts, such as in models of acute tissue injury and infection, highlighting its highly context-dependent nature (7–9). Specifically, necroptosis serves as a critical antiviral defense mechanism by eliminating infected cells, and its therapeutic activation offers a promising strategy to overcome apoptosis resistance in cancer therapy. Its involvement extends even to physiological processes such as reproduction, reflecting the functional versatility of this cell death modality (10). The molecular pathway of necroptosis has been most extensively characterized in the context of tumor necrosis factor (TNF) signaling. This canonical cascade involves sequential assembly of Complex I, the ripoptosome (Complex IIb), and ultimately the necrosome, wherein receptor-interacting protein kinase 1 (RIPK1) and RIPK3 phosphorylate mixed lineage kinase domain-like protein (MLKL), leading to plasma membrane permeabilization and cell lysis (11–13). While this framework provides an essential mechanistic foundation, recent studies have revealed a far more complex and dynamic regulatory landscape.

Beyond TNF, multiple stimuli can initiate necroptosis through alternative signaling axes. For example, viral nucleic acids can activate the cytosolic sensor Z-DNA binding protein 1 (ZBP1), while Toll-like receptor (TLR) engagement (particularly via TLR3 and TLR4), can utilize the adaptor protein TIR-domain-containing adapter-inducing interferon-β (TRIF) to trigger necroptosis, both converging on RIPK3-MLKL activation (14, 15). These non-canonical pathways expand the physiological and pathological scope of necroptosis and underscore its role as a versatile response to diverse cellular stresses. The activity and stability of the core necroptotic machinery: RIPK1, RIPK3, and MLKL, are tightly regulated through post-translational modifications such as phosphorylation and ubiquitination, as well as through interactions with a growing list of modulatory proteins including tripartite motif-containing 21, Akt, and heat−shock protein 90 (16–19). This intricate control network, coupled with extensive crosstalk between necroptosis and other cell death pathways (such as apoptosis, ferroptosis) and inflammatory signaling, introduces substantial complexity into predicting cellular outcomes.

Despite its well-established pathophysiological significance, a systematic synthesis of recent advances remains lacking, especially regarding non-canonical initiation routes and the multifaceted regulation of core components. This review therefore aims to (1): delineate the mechanisms and roles of non-canonical necroptosis pathways such as those mediated by ZBP1 and TRIF; (2) elucidate the complex regulatory landscape governing RIPK1, RIPK3, and MLKL; and (3) discuss emerging therapeutic strategies that target necroptosis in disease. By integrating these insights, we seek to provide a coherent framework that clarifies current understanding and identifies critical directions for future research and translational development.

## 2 Canonical and alternative pathways of necroptosis induction

### 2.1 TNF/TNF receptor 1 axis

**Figure 1:**
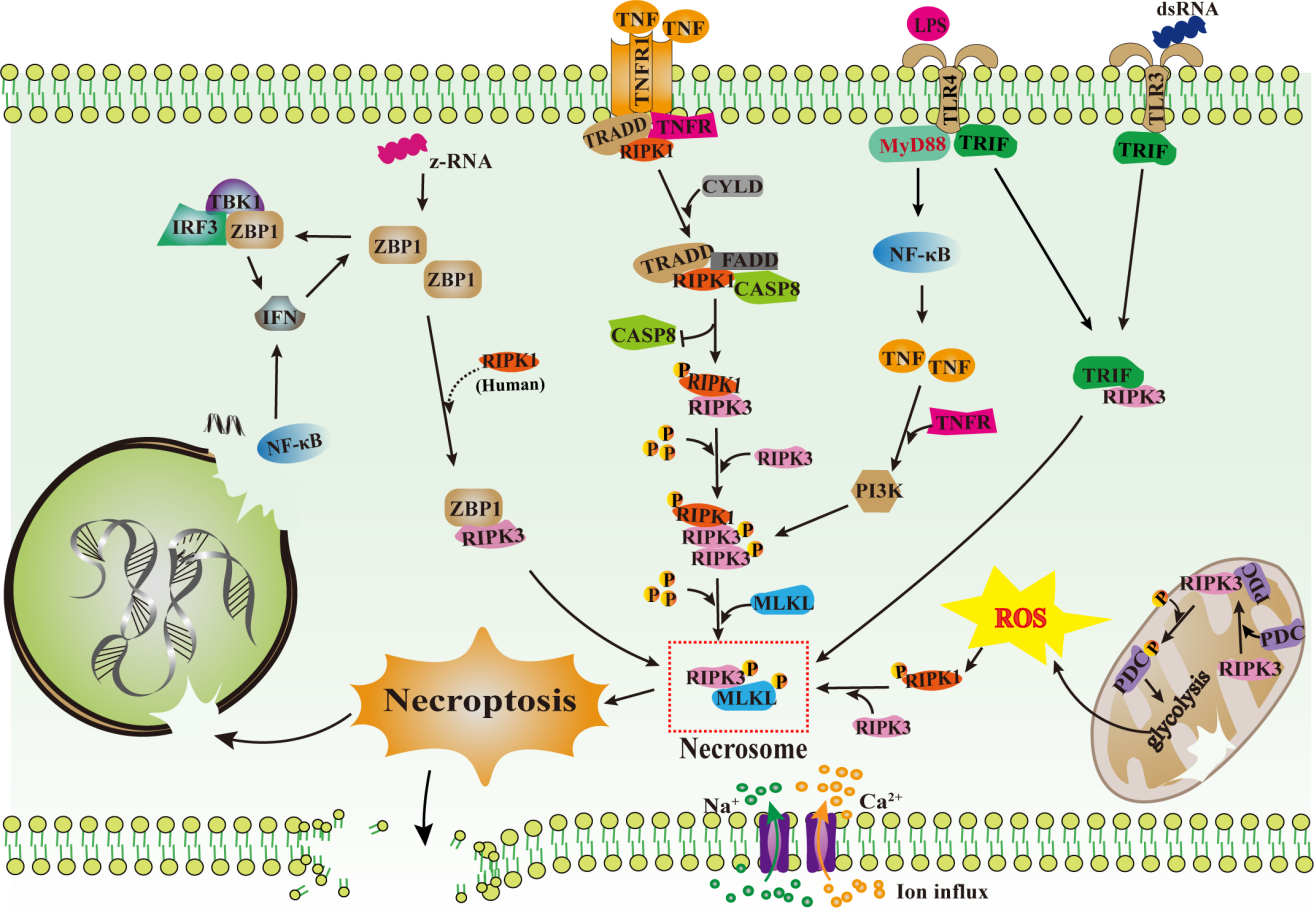
Overview of necroptosis signaling pathways. Necroptosis is executed via the RIPK3/MLKL axis. Three major routes lead to its activation: (1) TNFR1 engagement by TNF triggers the RIPK1/RIPK3/MLKL cascade; (2) ZBP1 senses viral z-RNA and recruits RIPK3; (3) TLR3 (dsRNA) and TLR4 (LPS) signal through TRIF, which directly binds RIPK3. In mitochondria, RIPK3 phosphorylates the pyruvate dehydrogenase complex (PDC) to modulate metabolic reprogramming. Upon activation, MLKL oligomerizes and translocates to plasma and nuclear membranes, causing membrane rupture, ion influx, and damage-associated molecular pattern release, thereby fueling inflammation and tissue damage. TNFR1 (TNF receptor 1), CYLD (cylindromatosis), CASP8 (Caspase-8), RHIM (RIPK homotypic interaction motif), TRIF (TIR-domain containing adapter including interferon-β), MyD88 (myeloid differentiation factor 88), IL-6 (interleukin-6), NF-κB (nuclear factor kappa-light-chain-enhancer of activated B cells), IFN (interferon), LPS (lipopolysaccharide), TBK1 (TANK-binding kinase 1), IRF3 (interferon regulatory factor 3), PDC (pyruvate dehydrogenase complex), mPTP (mitochondrial permeability transition pore), CaMKII (calcium-dependent protein kinase II).

Necroptosis is triggered by cytokine signaling, TLR engagement, and nucleic acid sensing pathways, with the TNF/TNF receptor 1 axis being the most extensively characterized (**Figure 1**). Mechanistically, TNF binding to TNF receptor 1 initiates the formation of complex I, followed by RIPK1 deubiquitination and assembly with FADD, caspase-8, and RIPK3 into ripoptosome (11). Under conditions permissive for necroptosis (e.g., low caspase-8, high RIPK3/MLKL expression), this complex further matures into the necrosome, wherein RIPK1 and RIPK3 undergo reciprocal phosphorylation (20). Activated RIPK3 then phosphorylates MLKL, inducing its oligomerization, membrane translocation, and pore formation, ultimately resulting in lytic cell death (21). This process releases damage-associated molecular pattern, thereby amplifying local and systemic inflammation and establishing a pathogenic feedback loop that exacerbates tissue injury (22). Given its role in driving inflammatory pathology, targeting the TNF-RIPK1/RIPK3/MLKL axis has emerged as a promising therapeutic strategy in conditions ranging from autoimmune diseases to severe viral infections such as SARS-CoV-2 (23).

### 2.2 ZBP1-mediated pathway

Beyond TNF, interferons (IFNs) and nucleic acid sensors constitute important inducers of necroptosis, particularly in the contexts of viral infection and genomic stress. IFN signaling can sensitize cells to necroptosis by upregulating key components of the pathway; for example, IFN-γ has been shown to enhance MLKL expression in lung epithelial cells, increasing their susceptibility to lytic death (24). Central to this IFN-driven response is ZBP1 (**Figure 1**), a cytosolic nucleic acid sensor equipped with Zα domains that recognize non-canonical Z-DNA and Z-RNA (25). Upon detecting viral Z-RNA or endogenous nucleic acids released under conditions of genomic instability, ZBP1 recruits RIPK3 via homotypic interaction motif (RHIM)-RHIM interaction, leading to RIPK3 autophosphorylation and subsequent MLKL activation (14, 26).

However, the requirement for RIPK1 in this process is fundamentally species−dependent. In human cells, ZBP1 cannot efficiently engage RIPK3 without RIPK1; instead, RIPK1 serves as an essential bridging adaptor that facilitates the formation of a stable and functional ZBP1/RIPK3 complex (27). In contrast, in murine cells, ZBP1 can directly bind and activate RIPK3, and RIPK1 plays an inhibitory role in this pathway (28). Consistent with this inhibitory function in mice, deficiency of RIPK1 or mutation of its RHIM domain promotes ZBP1−dependent necroptosis and inflammation *in vivo*, even though RIPK1 and ZBP1 do not directly interact under physiological conditions in mice (29). The ZBP1−mediated pathway has been implicated in various pathological conditions, such as diabetic nephropathy and inflammation (30). Moreover, caspase−6, though dispensable for canonical TNF−induced necroptosis, facilitates ZBP1/RIPK3 complex formation during influenza A virus infection, thereby potentiating necroptosis signaling and NLRP3 inflammasome activation (31).

The ZBP1 pathway is further modulated by mitochondrial factors. The pro-apoptotic protein PUMA can promote mitochondrial DNA release, which in turn activates ZBP1 and amplifies RIPK3/MLKL phosphorylation (32), illustrating a functional crosstalk between mitochondrial stress and nucleic acid-sensing pathways. Notably, PUMA itself can be upregulated in a RIPK3/MLKL-dependent manner during TNF-induced necroptosis, forming a positive feedback loop that enhances cell death sensitivity (33).

Given its critical role in antiviral defense and inflammation, the ZBP1/RIPK3/MLKL axis represents a promising target for therapeutic intervention. For instance, in severe SARS-CoV-2 infection, postmortem lung analyzes reveal pronounced necroptosis alongside apoptosis and immune infiltration (34). Co-treatment of the TNF and IFN-γ could activate the JAK/STAT1/IRF1 axis, induced nitric oxide production, drove Caspase-8/FADD-mediated and induced inflammatory cell death (35), highlighting the potential of combination cytokine inhibition in hyperinflammatory syndromes. These findings collectively underscore the complexity and pathophysiological relevance of ZBP1-mediated necroptosis across infectious, inflammatory, and metabolic disease contexts.

### 2.3 TRIF-mediated pathway

TLRs represent a widely expressed family of transmembrane receptors that play a central role in innate immunity and inflammatory signaling (36). Signal transduction downstream of TLRs is primarily mediated by the adaptor proteins myeloid differentiation primary response protein 88 (MyD88) and TRIF (**Figure 1**). Among TLRs, TLR3 and TLR4 utilize TRIF to initiate downstream signaling, which can culminate in necroptosis under conditions of caspase inhibition (15). This TRIF-dependent pathway has emerged as a critical non-canonical route for necroptosis induction, particularly in settings of infection or sterile inflammation.

The core necroptotic mediators RIPK1 and RIPK3 are also integral to TRIF-driven signaling. Upon caspase inhibition, ligands such as poly (I:C) (TLR3 agonist) or lipopolysaccharide (TLR4 agonist) promote the assembly of a necrosome via a TRIF/RIPK3 axis (37) (**Figure 1**). For example, lipopolysaccharide combined with caspase inhibition induces RIPK3-dependent necroptosis in macrophages, a process regulated by TLR4 dimerization and downstream adaptor recruitment (38, 39). The TRIF-mediated RIPK3/MLKL pathway has been implicated in diverse pathological contexts, including acute pancreatitis and dysregulated immune responses (40).

Mechanistically, RHIM that enables direct recruitment and activation of RIPK3, thereby bypassing RIPK1 in certain cellular contexts (3). Activated RIPK3 phosphorylates MLKL at key residues (such as Thr357/Ser358 in human MLKL), triggering its oligomerization, membrane translocation, and subsequent membrane disruption. Although TRIF can also engage RIPK1 to amplify inflammatory signaling (41), the relative contributions of RIPK1 versus RIPK3 in TLR-driven necroptosis remain context-dependent and are not fully resolved.

TLR4 signaling exhibits additional complexity due to its ability to engage both MyD88 and TRIF adaptors. While MyD88 typically drives early NF-κB activation, it can also indirectly promote RIPK1/RIPK3 activation by inducing autocrine/paracrine TNF (42). This creates a feed-forward loop linking TLR4 activation to necroptotic amplification. Pharmacological inhibition of TLR4 (e.g., using the TLR4 antagonist factor-β-activated kinase-242) has been shown to reduce RIPK3 expression, NF-κB activation, and necroptosis in experimental models of acute pancreatitis, underscoring the therapeutic potential of modulating this pathway (43). Research has also demonstrated that pharmacological inhibition of RIPK3 in cardiomyopathy and neurological disorders can influence the TLR4/MyD88 pathway, suggesting the complex regulatory interplay between TLR4 and RIPK3 in disease pathogenesis (44, 45). Emerging regulatory layers further fine-tune TLR-mediated necroptosis. MicroRNAs dynamically regulate expression of TLR4 and its downstream signaling components (such as MyD88, NF-κB), thereby modulating inflammatory and necroptotic responses (46). However, the translational utility of these regulators in clinical diagnosis or monitoring remains limited by challenges in tissue accessibility and dynamic expression profiles.

Notably, TLR4 signaling engages in extensive crosstalk with other forms of regulated cell death. Intravital imaging of cardiac transplants revealed that ferroptosis orchestrates initial neutrophil recruitment through a TLR4/TRIF/type I IFN axis, while necroptosis drives a subsequent wave of cell death that amplifies the inflammatory response initiated by ferroptosis (47). These findings highlight the multimodal nature of inflammatory cell death and the intricate interplay between distinct lethal subroutines.

### 2.4 Other emerging triggers and context-specific circuits

Beyond TLR- and cytokine- driven pathways, recent studies have identified additional triggers and context-specific circuits that contribute to necroptosis. In myocardial ischemia/reperfusion injury, RIPK3 activates Ca²^+^/calmodulin-dependent protein kinase II (CaMKII), leading to mitochondrial permeability transition pore opening, a pathway designated RIPK3/CaMKII/mitochondrial permeability transition pore axis (48). The research further extends this mitochondrial connection by demonstrating that RIPK3 phosphorylates mitofusin-2, which promotes endoplasmic reticulum-mitochondria tethering and facilitates mitochondrial calcium overload, ultimately driving necroptotic cell death (49). Similarly, inhaled hydrogen postconditioning can attenuate skin ischemia/reperfusion injury via a RIPK/MLKL/phosphoglycerate mutase family member 5 dependent mechanism (50). In the context of antitumor immunity, the RIPK3/RIPK1/NF-κB axis appears dispensable for optimal CD8^+^ T-cell cross-priming induced by necroptotic cells, suggesting immune-contextual specificity in signaling outcomes (51).

These discoveries illustrate the expanding landscape of necroptosis triggers and the tissue-specific regulation of its execution. However, current understanding of many alternative pathways remains preliminary, often constrained by model systems, cell-type specificity, and triggering conditions. Further mechanistic and translational studies are needed to elucidate their physiological relevance, regulatory networks, and therapeutic potential in human diseases.

Importantly, the role of necroptosis in oncology has garnered increasing attention in recent years. Beyond the contexts described above, necroptosis has been implicated in the regulation of the tumor immune microenvironment, where necroptotic tumor cells can release damage-associated molecular patterns to promote immunogenic cell death and activate anti-tumor immunity (52). Conversely, sustained necroptotic signaling may contribute to therapy resistance in certain cancers, and emerging strategies seek to harness necroptosis induction as a novel anti-cancer approach (53, 54). These findings highlight the dual, context-dependent functions of necroptosis in tumor initiation, progression, and therapeutic response, underscoring its potential as both a prognostic biomarker and a therapeutic target.

## 3 Multifaceted regulation of the core necroptotic machinery

**Figure 2:**
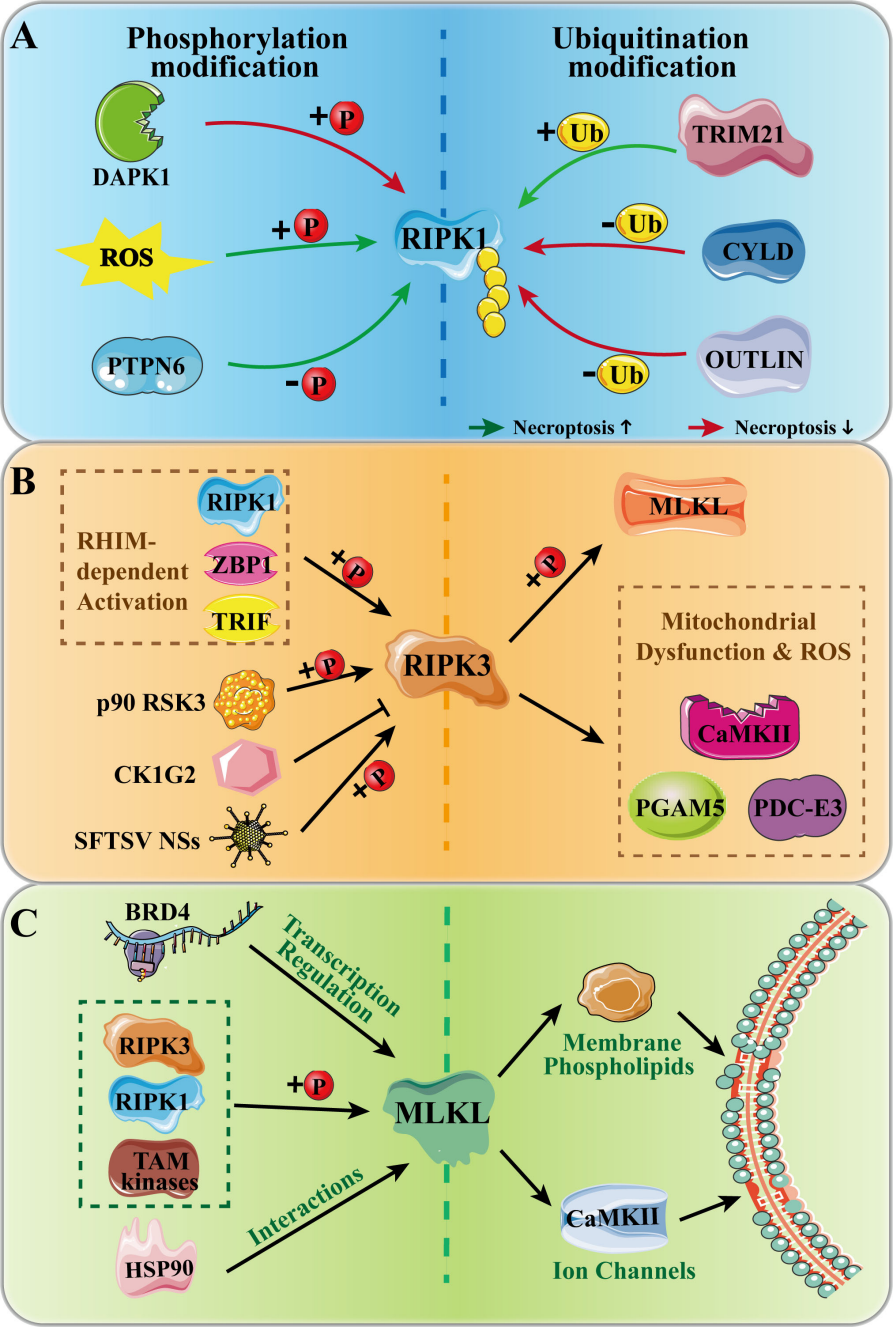
Multilayered regulation of RIPK1, RIPK3, and MLKL mediates the execution of necroptosis. Upstream signals converge on RIPK1, which integrates survival and death cues. RIPK3 then amplifies and disseminates the death signal by phosphorylating both the executor MLKL and diverse substrates linking necroptosis to mitochondrial dysfunction and metabolic reprogramming. MLKL itself is regulated at transcriptional, post-translational, and membrane-localization levels. The dynamic balance of these regulatory layers determines the threshold for inflammatory cell death.

The necroptosis signaling network is characterized by intricate and multilayered regulation, ensuring precise spatiotemporal control over this potent form of inflammatory cell death. The core mediators, RIPK1, RIPK3, and MLKL, function as central hubs where diverse upstream signals converge, thereby determining the cellular fate (55). Moving beyond the canonical phosphorylation cascade, their activities are subjected to stringent and sophisticated regulation through a spectrum of post-translational modifications, dynamic protein-protein interactions, and transcriptional and epigenetic mechanisms (**Table 1**, **Figure 2**). This elaborate regulatory architecture ensures that necroptosis is deployed in a highly context-dependent manner, preventing unwarranted cell death.

**Table 1:** Regulators of key necroptosis mediators and their roles in disease.

Mediator	Regulator	Mechanism	Disease	Ref.
RIPK1	Death-associated protein kinase 1	Death-associated protein kinase 1 inhibits RIPK1 activity by directly phosphorylating the RIPK1 protein.	Tumors	(59)
	ROS	ROS promote necroptosis by inducing RIPK1 autophosphorylation.	Septic cardiomyopathy, viral myocarditis	(17, 60)
	Tyrosine phosphatase-6	Tyrosine phosphatase-6 primarily dephosphorylates tyrosine residues on the RIPK1 protein through its phosphatase activity.	Tumors, autoimmune and Inflammatory Diseases	(61, 111)
	PI3K	The PI3K/Akt pathway suppresses RIPK1−dependent necroptosis by phosphorylating and inhibiting RIPK1 kinase activity.	Tumors, systemic inflammation, and myocardial ischemia/reperfusion injury	(62, 63)
	Tripartite motif containing 21	The tripartite motif containing 21 directly interacts with RIPK1 and catalyzes the ubiquitination of RIPK1 through its RING domain.	Viral infections and antiviral Immunity, autoimmune diseases	(19, 66)
	Cylindromatosis	Cylindromatosis modulates the ubiquitination status of RIPK1.	The pathogenesis of neuroinflammatory and neurodegenerative diseases, systemic hyperinflammatory syndromes, etc.	(57, 58)
	OTULIN	During necroptosis, phosphorylation of OTULIN at Tyr-56 regulates RIPK1 ubiquitination and drives cell death.	Inflammation	(64, 65)
RIPK3	Caspase-6	Caspase-6 promotes the inflammatory response triggered by RIPK3 binding to ZBP1 through interaction with RIPK3.	Viral infectious disease	(31)
	ZBP1	ZBP1 directly recruits and binds to RIPK3 through its RHIM domain, leading to RIPK3 autophosphorylation and activation.	Viral infections, inflammatory diseases, reproductive system disorders, etc.	(112–114)
	TRIF	TRIF can directly bind to the RHIM domain of RIPK3, thereby directly activating the downstream necroptosis pathway.	Infections, inflammation-related damage, tumors, etc.	(37, 115)
	p90 ribosomal protein S6 kinase 3 (RSK3)	RSK3 directly acts as an upstream regulator of RIPK3 phosphorylation, promoting the activation of the necroptosis pathway.	Ischemic retinal injury	(68)
	Casein kinase 1G2	Casein kinase 1G2 directly binds to RIPK3 to inhibit its kinase activity and downstream necroptosis signaling.	Male reproductive aging	(10)
	Calcium-dependent protein kinase II (CaMKII)	RIPK3 can directly phosphorylate and activate CaMKII, thereby triggering necroptosis or suppressing excitotoxicity to exert neuroprotective effects.	Myocardial ischemia/reperfusion injury, heart failure, neurological injury	(116, 117)
	Pyruvate dehydrogenase complex (PDC)	RIPK3 phosphorylates threonine 135 on the E3 subunit of PDC, thereby enhancing PDC catalytic activity.	Cancer	(71, 72)
	Phosphoglycerate mutase family 5	Phosphoglycerate mutase family member 5 is activated by RIPK3 phosphorylation, which in turn dephosphorylates Drp1, promoting mitochondrial fission and amplifying the death signal.	Ischemia-reperfusion injury, neurodegenerative diseases	(73)
MLKL	Bromodomain protein 4	Bromodomain protein 4 positively regulates necroptosis by transcriptionally controlling MLKL expression.	Inflammation-related diseases	(77, 78)
	Heat shock protein 90	Heat shock protein 90 binds to MLKL, preventing its degradation by the proteasome and promoting MLKL activation and membrane translocation.	Inflammatory and infectious diseases	(18, 79)
	RIPK1	RIPK1 can directly phosphorylate MLKL under specific conditions (particularly in mouse cells).	Inflammation model	(118)
	TAM kinase (Tyro3, Axl, Mer)	During phagocytosis, TAM kinases are activated by exposed phosphatidylserine and can directly phosphorylate MLKL.	Inflammation-related diseases	(81)

### 3.1 Regulation of RIPK1: the decision-making integrator

As the pivotal node where survival and death signals converge, RIPK1 functions as a molecular integrator whose activity and downstream cell fate are governed by multilayered regulatory mechanisms (56). Its function is dynamically shaped by the antagonistic balance of activating and inhibitory signals, primarily through phosphorylation and ubiquitination modifications (**Table 1**). Among these, the most well-established core regulatory nodes include: RHIM domain-mediated interaction with RIPK3, which is essential for necrosome formation and subsequent necroptotic execution; caspase-8-mediated cleavage of RIPK1, which serves as a critical checkpoint to prevent aberrant necroptosis by removing its kinase domain; and cylindromatosis-mediated deubiquitination, which facilitates RIPK1 release from pro-survival Complex I and promotes its incorporation into the pro-death necrosome (57, 58).

In addition to these core nodes, several well-validated mechanisms further fine-tune RIPK1 activity. Phosphorylation serves as a crucial molecular switch for RIPK1 activity, with the balance between specific kinases and phosphatases determining the cellular outcome. Death-associated protein kinase 1-mediated phosphorylation at Ser321 represents a well-validated inhibitory mechanism, directly suppressing RIPK1’s pro-necrotic function (59). In contrast, ROS promote necroptosis by inducing RIPK1 autophosphorylation, establishing a feedforward loop that amplifies cell death signaling (17, 60). However, the precise molecular details of ROS-induced autophosphorylation, including the specific phosphorylation sites involved and the upstream sources of ROS, remain incompletely characterized and warrant further investigation. Phosphatases that dephosphorylate RIPK1 primarily provide negative regulation, with protein tyrosine phosphatase-6 representing another well-validated inhibitory node (61). Furthermore, upstream signaling pathways converge on RIPK1 to fine-tune the necroptotic threshold. The pro-survival PI3K/Akt pathway, for instance, negatively regulates classical RIPK1-dependent necroptosis by ultimately inhibiting RIPK1 kinase activity, illustrating how integrated cellular networks modulate cell death sensitivity (62, 63).

Ubiquitination constitutes another essential layer of control, precisely regulating RIPK1’s stability, activity, and recruitment into signaling complexes. This reversible modification is dynamically governed by the opposing actions of specific ligases and deubiquitinases. Conversely, the linear deubiquitinase OTULIN counteracts cylindromatosis-mediated deubiquitination; phosphorylation of OTULIN at Tyr-56 during necroptosis regulates RIPK1 ubiquitination to drive cell death, though the precise functional interplay between OTULIN and cylindromatosis in different cellular contexts remains an area of active investigation (64, 65). E3 ligases, such as tripartite motif-containing 21, generally act as positive regulators by directly interacting with RIPK1 and catalyzing its ubiquitination, thereby promoting its activation and pro-necrotic function (19, 66). However, it should be noted that the specific ubiquitin chain types generated by tripartite motif-containing 21 and their differential contributions to RIPK1 stability versus signaling activity represent an unresolved question in the field.

The dynamic equilibrium between these ubiquitinating and deubiquitinating enzymes, together with the opposing actions of kinases and phosphatases, ensures that RIPK1-mediated cell fate decisions are made in a highly context-dependent manner. Importantly, these regulatory layers do not operate in isolation; phosphorylation events can influence ubiquitination status and vice versa. For example, specific phosphorylation of RIPK1 can create docking sites for E3 ligases or deubiquitinases, while ubiquitination can affect the accessibility of phosphorylation sites. This integrated signaling code, rather than any single modification, ultimately determines whether RIPK1 promotes survival or executes necroptosis. Consequently, the RIPK1 signaling node represents a highly attractive target for therapeutic intervention in necroptosis-associated diseases.

### 3.2 Regulation of RIPK3: the core executor of necroptosis

RIPK3 is the central kinase in the execution phase of necroptosis, forming the essential RIPK3-MLKL module that drives cell death (67). Its activation and signaling output are modulated by a diverse array of kinases, phosphatases, and downstream substrates (**Table 1**), which confer context-dependent regulation and expand its pathophysiological roles.

The most well-established mechanism for RIPK3 activation involves its direct engagement by innate immune sensors via RHIM domain interactions. Beyond its well-characterized interaction with RIPK1, RIPK3 can be activated by ZBP1 and TRIF through their respective RHIM domains, leading to RIPK3 autophosphorylation and the initiation of necroptosis (14, 15). This mode of activation has been extensively validated across diverse experimental systems, including viral infection and sterile inflammation models.

In addition to RHIM-mediated activation, RIPK3 can also be activated by upstream kinases such as p90 ribosomal protein S6 kinase 3 (RSK3), which has been shown to promote necroptosis in ischemic retinal injury by directly phosphorylating RIPK3 (68). This represents a context-specific activation mechanism, though its broader relevance across different tissues remains to be determined. Conversely, negative regulation of RIPK3 is exemplified by casein kinase 1G2, which suppresses RIPK3 kinase activity and attenuates necroptosis, a mechanism linked to testicular aging (10). Furthermore, emerging evidence reveals that certain pathogens can bypass conventional RHIM-dependent signaling: for instance, the nonstructural protein NSs of severe fever with thrombocytopenia syndrome virus binds directly to the kinase domain of RIPK3, promoting its autophosphorylation and necroptosis activation independently of RHIM interactions (69). Together, these studies illustrate that the activation threshold and signaling output of RIPK3 are finely tuned by a variety of context-specific regulators, reflecting the adaptability of necroptosis in diverse pathological settings.

Beyond phosphorylating MLKL, RIPK3 executes necroptosis through a repertoire of key substrates that link cell death signaling to broader cellular dysfunction. The most well-established downstream effector is calcium/calmodulin−dependent protein kinase II (CaMKII), which is directly phosphorylated and activated by RIPK3, leading to mitochondrial dysfunction and contributing to myocardial ischemia/reperfusion injury, heart failure, and neurological damage (48, 70). Additional substrates further expand RIPK3’s functional repertoire by linking necroptosis to metabolic and structural alterations. In cancer−associated metabolic reprogramming, RIPK3 phosphorylates the pyruvate dehydrogenase complex (PDC) E3 subunit at threonine 135, enhancing its catalytic activity and influencing tumor progression (71, 72). While this mechanism has been elegantly demonstrated, its broader relevance across different disease contexts remains an open question. Furthermore, RIPK3−activated phosphoglycerate mutase family member 5 promotes mitochondrial fission, amplifying necroptosis signaling in conditions such as ischemia−reperfusion injury and neurodegenerative diseases (73). Through these diverse substrates, RIPK3 links necroptosis signaling to metabolic, structural, and functional alterations within the cell.

Collectively, the regulation of RIPK3 represents a layered system in which core activation nodes, such as RHIM-mediated engagement by ZBP1 and TRIF, are supplemented by context-specific modulators and downstream effectors that amplify or diversify the death signal. The relative contributions of these distinct pathways to overall necroptotic cell death remain an important area for future investigation.

### 3.3 Regulation of MLKL: the terminal effector

While MLKL oligomerization and membrane pore formation are central to necroptotic execution (5), its activation is governed by multiple regulatory layers (**Table 1**). The most well-established core regulatory node is RIPK3-mediated phosphorylation of MLKL at the activation loop (e.g., Thr357/Ser358 in human MLKL), which triggers its conformational change, oligomerization, and membrane translocation (74). Pharmacologically, this core mechanism is targetable; for instance, the salt-inducible kinase inhibitor HG-9-91–01 suppresses necroptosis by disrupting RIPK3/MLKL interaction (75, 76). Beyond this core mechanism, additional layers of regulation fine-tune MLKL expression and activity. Bromodomain protein 4 acts as a transcriptional regulator by binding to the MLKL gene promoter to promote its expression (77, 78). At the protein level, heat shock protein 90 interacts with MLKL, protecting it from proteasomal degradation and facilitating its activation and membrane translocation (18, 79). In addition to RIPK3, other kinases can further fine-tune MLKL activity in specific contexts (80). During phagocytosis, TAM kinases (Tyro3, Axl, Mer) are activated by exposed phosphatidylserine and can also directly phosphorylate MLKL, linking phagocytic cues to necroptotic execution (81). This layer of regulation is also pharmacologically targetable. For example, the anesthetic ketamine suppresses necroptosis by reducing ROS-dependent MLKL phosphorylation (75, 76).

Mechanistically, MLKL-mediated membrane disruption exhibits context-dependent pathways. The canonical pathway involves MLKL oligomerization, membrane translocation, and pore formation via binding to phosphatidylinositol lipids and cardiolipin. However, non-canonical routes exist. For example, in a neuroinflammatory model of glaucoma, TNF induces a specialized form of axonal degeneration with necroptosis-like features, but independent of MLKL’s direct pore-forming function (82). Moreover, evidence suggests MLKL may sometimes compromise membrane integrity indirectly, such as through activation of ion channels like TRPM7 (83). These observations collectively indicate that the terminal execution phase of necroptosis is more heterogeneous than previously appreciated, with MLKL-independent mechanisms contributing to cell death in certain contexts.

As research advances, an expanding repertoire of factors has been identified that directly or indirectly modulate the RIPK1-RIPK3-MLKL axis at multiple levels. The intricate interplay among these regulators, ranging from core transcriptional and post-translational control nodes to context-specific modulators and controversial mechanisms, not only underscores necroptosis as a highly tunable and context-dependent cell death pathway but also reveals novel nodes for potential therapeutic intervention in a wide spectrum of necroptosis-associated diseases.

## 4 Crosstalk with other cellular processes

**Figure 3:**
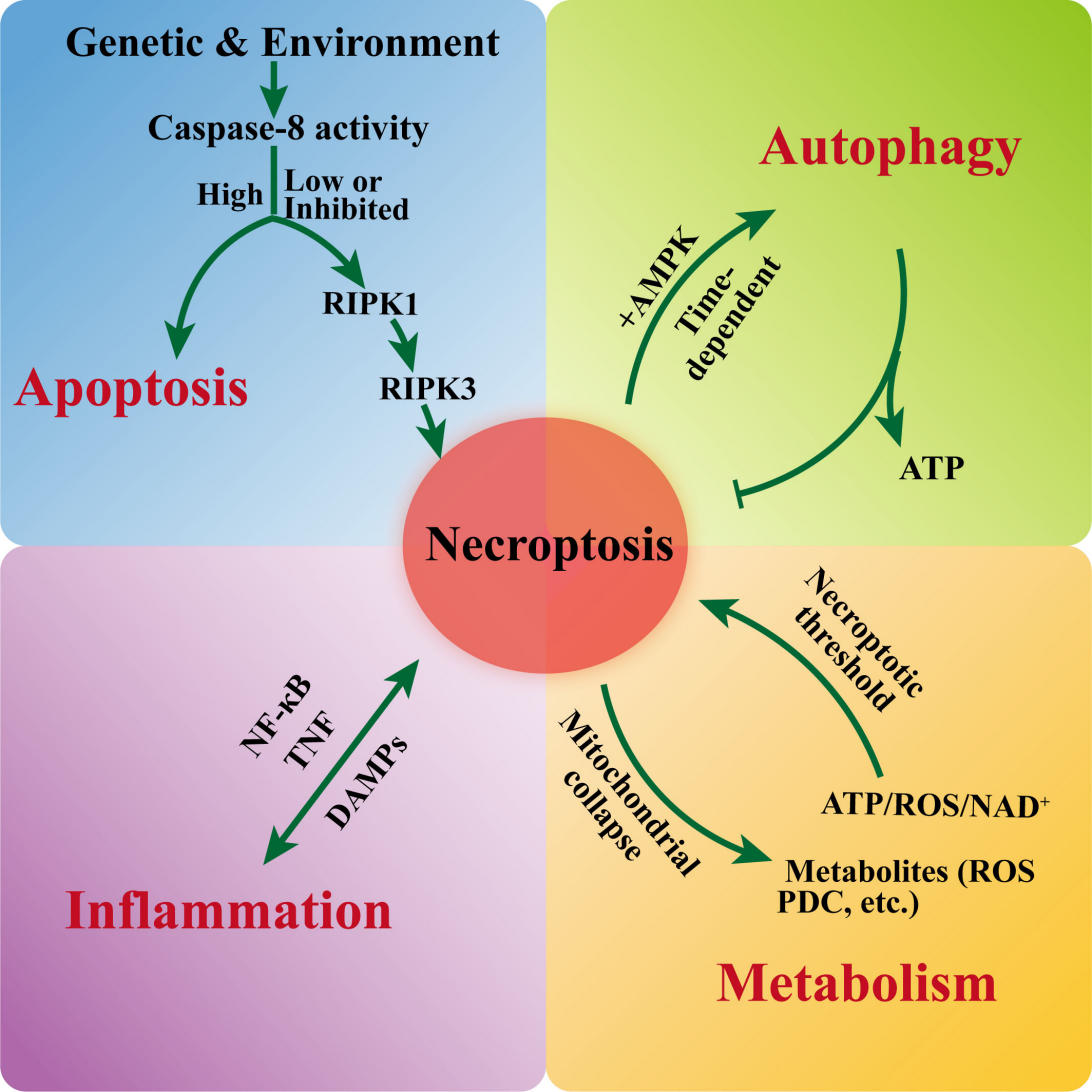
Interactions between necroptosis and apoptosis, autophagy, inflammation, and metabolic signaling under different conditions. NF-κB (nuclear factor kappa-light-chain-enhancer of activated B cells), PDC (pyruvate dehydrogenase complex), AMPK (AMP-activated protein kinase), DAMP (damage-associated molecular pattern).

Necroptosis is not an isolated pathway but operates within an extensive network of molecular crosstalk with other cellular processes, including apoptosis, autophagy, inflammatory signaling, and metabolic regulation (**Figure 3**). These interactions enable cells to integrate diverse stress signals and determine death modalities in a context-dependent manner, thereby shaping physiological outcomes and disease pathogenesis (52).

### 4.1 Apoptosis

Necroptosis and apoptosis share common upstream signaling components and are tightly regulated by genetic and environmental cues, allowing cells to switch between these two death modalities depending on cellular context and stress intensity (84, 85) (**Figure 3**). For instance, *Angiostrongylus* cantonensis infection stimulates microglial TNF release, which concurrently triggers astrocyte apoptosis and neuronal necroptosis, illustrating how the same stimulus can elicit distinct death pathways in different cell types (86). Similarly, in intestinal epithelial models, high concentrations of *C. perfringens* enterotoxin induce necroptosis, whereas lower concentrations promote apoptosis; notably, inhibition of MLKL oligomerization with calpain inhibitors shifts the death mode toward apoptosis (87), suggesting a reciprocal regulatory relationship between these pathways. Although the precise molecular switches governing the apoptosis–necroptosis transition remain incompletely defined, key candidates include caspase-8 activity levels, the cellular concentration of cFLIP isoforms, and the phosphorylation status of RIPK1 (88–90). Accordingly, high Caspase-8 activity promotes apoptosis, whereas low or inhibited caspase-8 activity relieves the Caspase-8-mediated suppression of the RIPK1/RIPK3 axis, thereby enabling necroptosis execution. Further studies are needed to elucidate how these factors integrate environmental and metabolic cues to guide cell fate decisions.

### 4.2 Autophagy

The interplay between necroptosis and autophagy is bidirectional and phase-dependent (**Figure 3**). Under conditions of energy stress, autophagy can rescue cells by restoring ATP levels, and its inhibition may precipitate a metabolic crisis that promotes necroptosis (52). Interestingly, TNF-induced necroptosis initiates early autophagy through RIPK3-dependent activation of AMP-activated protein kinase, which may initially support cell survival or facilitate death signaling (91). However, at later stages, necroptosis signaling suppresses autophagic flux, possibly to prevent the clearance of damaged organelles and thus amplify cell death. This temporal regulation highlights the nuanced and context-specific nature of autophagy-necroptosis crosstalk, which may vary across cell types and stress conditions.

### 4.3 Inflammation

Necroptosis is both a consequence and a driver of inflammation, engaging in feed-forward loops with inflammatory signaling pathways, particularly NF-κB (**Figure 1**). NF-κB activation can induce the expression of TNF and other cytokines, thereby sensitizing cells to necroptosis (92). In turn, necroptotic cell release damage-associated molecular patterns and inflammatory mediators that further activate NF-κB and other pro-inflammatory cascades, exacerbating tissue injury in conditions such as ischemic organ damage, neurodegenerative diseases, and inflammatory bowel disease. Environmental toxins such as cadmium and lead have been shown to promote necroptosis via NF-κB dependent mechanisms, whereas protective agents like selenium can attenuate cell death by inhibiting the MAPK/NF-κB axis (93, 94). These findings underscore the therapeutic potential of modulating inflammatory signaling to control necroptosis in environmental and disease contexts. However, it is important to recognize that necroptosis also plays a beneficial role in host defense. For instance, necroptotic elimination of virus-infected cells limits viral spread and contributes to pathogen clearance, as demonstrated in influenza A virus and SARS-CoV-2 infections (31, 35). This dual nature, pathogenic when dysregulated but protective under controlled conditions, underscores the need for context-specific therapeutic strategies that preserve host defense while curbing excessive inflammation.

### 4.4 Metabolism

Mitochondria serve as critical hubs for necroptosis signaling and metabolic crosstalk (80) (**Figure 3**). The necrosome can induce mitochondrial dysfunction through mechanisms including mitophagy, mitochondrial ROS production, and permeability transition, all of which amplify cell death (95). Conversely, metabolic state influences necroptosis sensitivity (2). For example, inhibition of the mitochondrial pyruvate carrier by UK5099 suppresses necroptosis, highlighting the role of mitochondrial metabolism in regulating this pathway (71). Additionally, RIPK3 has been shown to phosphorylate metabolic enzymes such as the pyruvate dehydrogenase complex (71, 72), linking necroptosis signaling directly to cellular metabolism and suggesting that metabolic reprogramming may either promote or restrain necroptosis depending on the bioenergetic context.

Beyond metabolic regulation, emerging evidence indicates that necroptosis does not operate in isolation but rather integrates with other programmed cell death pathways through higher-order signaling complexes. Recent studies propose that necroptosis, apoptosis, and pyroptosis can be co-activated through such complexes, termed “PANoptosomes”, which incorporate components such as RIPK1, RIPK3, FADD, caspase-8, and NOD-like receptor family pyrin domain-containing 3 (96, 97). The assembly of PANoptosomes is typically initiated by specific triggers, including viral nucleic acids, bacterial components, or cytokines, which engage upstream sensors to nucleate the complex. Of particular relevance to the necroptosis pathways discussed earlier, the nucleic acid sensor ZBP1 has been shown to nucleate a PANoptosome that simultaneously activates RIPK3/MLKL-mediated necroptosis, caspase-8-dependent apoptosis, and inflammasome-driven pyroptosis during viral infection or cytokine storm (96). This provides a direct mechanistic link between the ZBP1-RIPK3-MLKL axis and the broader PANoptosis framework, illustrating how necroptotic signaling integrates with other death modalities within a single supramolecular complex. Beyond ZBP1, other sensors such as AIM2 can also form PANoptosomes in response to distinct pathogens (98).

Such coordinated activation mounts a multi−faceted inflammatory response with important implications for understanding complex immunopathology. Beyond viral infection, PANoptosis has been implicated in a range of pathological conditions, including bacterial sepsis, inflammatory bowel disease, and neurodegenerative disorders, where dysregulated activation of the PANoptosome contributes to excessive inflammation and tissue damage (96, 97). The recognition that multiple cell death pathways converge at the level of PANoptosomes reframes cell death crosstalk from parallel pathways into an integrated signaling network offering a more effective therapeutic strategy that targets shared upstream sensors or core components, rather than inhibiting individual death pathways alone.

Collectively, these findings indicate that necroptosis is embedded within a broader signaling network influenced by metabolic status, inflammatory, and interconnected cell death pathways. Its functional outcomes, whether pathogenic or protective, are highly context-dependent, and are shaped by dynamic interactions with apoptosis, autophagy, and key regulators such as NF-κB. Although significant progress has been made in mapping these connections, the molecular details underlying pathway switching, compensatory mechanisms, and tissue-specific variations remain incompletely understood. Further elucidation of these regulatory circuits will not only advance fundamental knowledge of cell death integration but also provide a rational basis for targeting necroptosis in a wide range of pathological conditions, from cancer and neurodegeneration to infectious and inflammatory diseases.

## 5 Therapeutic implications and future perspectives

Despite the growing recognition of necroptosis as a pivotal contributor to acute and chronic pathologies, the molecular complexity and contextual regulation of its signaling network remain incompletely elucidated. Current methodological approaches to study necroptosis predominantly rely on pharmacological inhibition, using compounds such as Necrostatin-1 (RIPK1 inhibitor) or necrosulfonamide (MLKL inhibitor), in conjunction with the assessment of key molecular markers, including phosphorylated MLKL, RIPK3 oligomerization, and membrane integrity assays (99). However, the persistent unmet clinical need in managing hyperinflammatory states, as starkly evidenced during the SARS-CoV-2 pandemic, highlights the urgency to precisely define the pathogenic versus protective roles of necroptosis across different disease contexts (100).

Targeting the core molecular machinery of necroptosis has spurred the development of diverse therapeutic agents. Clinically, biologic agents that indirectly modulate necroptosis susceptibility, such as TNF inhibitors (e.g., etanercept, infliximab, adalimumab), are widely used and FDA-approved for autoimmune and inflammatory conditions including rheumatoid arthritis, inflammatory bowel disease, psoriasis, and ankylosing spondylitis (101–104). These agents primarily block upstream death receptor signaling, thereby suppressing both apoptotic and necroptosis pathways. In parallel, a new generation of direct necroptosis inhibitors is advancing in preclinical development. These include selective RIPK1 inhibitors (Necrostatin-1), RIPK3 inhibitors (GSK’840, GSK’872, Zharp-99), MLKL inhibitors (necrosulfonamide, bromodomain inhibitors targeting bromodomain protein 4) (105, 106). Such compounds have demonstrated efficacy in reducing tissue injury and inflammation in models of ischemic organ damage, neurodegenerative disease, and sterile inflammation.

Beyond conventional pharmacology, non-pharmacological interventions and endogenous peptide modulators have also shown promise in regulating necroptosis. For example, electroacupuncture in rodent models of intracerebral hemorrhage was reported to attenuate RIPK3/MLKL-mediated necroptosis and improve neurological outcomes (107). Similarly, treatment with the endogenous peptide apelin in streptozotocin-induced diabetic rats reversed hippocampal necroptosis, alleviated cognitive impairment, and preserved neuronal viability (108). While these findings underscore the therapeutic potential of modulating necroptosis through multiple modalities, most evidence remains confined to preclinical studies, and translational validation regarding efficacy, safety, and patient stratification is critically needed.

Inhibition of necroptosis represents a promising therapeutic strategy, particularly in diseases driven by lytic cell death and sterile inflammation. Preclinical studies consistently indicate that dampening necroptosis can reduce systemic inflammation, limit tissue damage, and improve functional recovery (109). However, major pharmacological challenges persist. Many existing necroptosis inhibitors exhibit moderate selectivity, suboptimal pharmacokinetic profiles, off-target effects, or dose-limiting toxicities, factors that have largely restricted their use to experimental settings (110).

Beyond the development of improved inhibitors, the therapeutic landscape of necroptosis is characterized by a fundamental duality: inhibition to curb excessive inflammation versus induction to eliminate apoptosis-resistant cancer cells. As mentioned earlier, accumulating evidence supports the anticancer potential of necroptosis induction, with preclinical studies demonstrating efficacy in colorectal cancer, melanoma, and other malignancies (52–54). This dual therapeutic paradigm, suppressing necroptosis in chronic inflammatory conditions while activating it in oncology, underscores the critical importance of context-dependent modulation. However, successful clinical translation will require not only improved pharmacological agents but also robust biomarkers to stratify patients based on disease context and necroptotic activity. Looking forward, deeper mechanistic studies are needed to uncover isoform-specific, tissue-selective, or context-dependent regulatory nodes, which will inform the rational design of next-generation therapeutics and enable precision patient stratification. The inherent complexity and context-dependency of necroptotic networks, driven by diverse triggers, multifunctional signaling molecules, and extensive crosstalk with other cell-death and inflammatory pathways, continue to pose challenges for therapeutic exploitation, yet also offer rich opportunities for precision intervention.

## 6 Conclusions

This review has synthesized current knowledge on both canonical and atypical necroptosis pathways. These parallel signaling routes converge on RIPK3-mediated MLKL activation while responding to distinct stimuli (e.g., viral nucleic acids and Toll-like receptor signaling), thereby expanding the physiological and pathological contexts of necroptosis beyond TNF-driven inflammation. By further integrating emerging evidence on the transcriptional, post-translational, and contextual regulation of necroptotic components, as well as their dynamic crosstalk with apoptosis, autophagy, and metabolic networks, we underscore how necroptosis functions as a versatile and tightly controlled cell death modality that can either promote or limit disease progression depending on context. Its ability to eliminate infected cells and apoptosis-resistant tumors highlights its protective and therapeutic potential, complementing its well-recognized pathogenic roles.

The inherent biological complexity of necroptosis, characterized by multifunctional signaling molecules, extensive crosstalk with apoptosis, autophagy, and inflammatory pathways, and highly context-dependent outcomes, continues to pose significant challenges for therapeutic exploitation. To advance the field toward clinical translation, future research maybe should prioritize several key directions: 1) developing more specific and clinically viable inhibitors; 2) establishing context-dependent biomarkers to differentiate necroptotic activity originating from distinct upstream triggers; 3) elucidating the differential roles of necroptosis in various tissues and disease stages; and 4) exploring combination therapies that target necroptosis in concert with complementary pathways.

As our understanding of necroptosis evolves from a linear death pathway to a multi-input signaling network, the opportunities for therapeutic exploitation grow accordingly. A nuanced, context-aware approach to targeting necroptosis—whether by inhibiting its execution in degenerative diseases or activating it in cancer—will be essential for translating mechanistic insights into safe and effective treatments across a spectrum of human diseases.
